# Genetic profiling of CLL: a ‘TP53 addict' perspective

**DOI:** 10.1038/cddis.2015.415

**Published:** 2016-01-14

**Authors:** L Lodé, F Cymbalista, T Soussi

**Affiliations:** 1 Haematology Laboratory, UMR-CNRS-5235, CHU, F-34000 Montpellier, France; 2 INSERM U978, Bobigny, France; 3 Université Paris 13, Sorbonne Paris Cité, Labex ‘Inflamex', Bobigny, France; 4 Service d'Hématologie Biologique, Hôpital Avicenne, AP-HP, Bobigny, France; 5 Sorbonne Université, UPMC Univ Paris 06, F- 75005 Paris, France; 6 Department of Oncology-Pathology, Karolinska Institutet, Cancer Center Karolinska (CCK) R8:04, Stockholm SE-171 76, Sweden; 7 INSERM, U1138, Centre de Recherche des Cordeliers, Paris, France

Chronic lymphocytic leukaemia (CLL) is a B-cell malignancy with a highly variable clinical course. Whereas some patients require treatment relatively soon after diagnosis, others may stay free of symptoms for many years with a standard ‘watch and wait' surveillance approach. Genomic analysis of asymptomatic patients and patients with progressive or relapsed refractory disease makes CLL an excellent model to study the course of genetic modifications in relation to the pathobiology of this disease.

The use of karyotype analysis first demonstrated that the deletions of chromosomes 13q, 17p, and 11q as well as trisomy 12 were recurrent aberrations in CLL. Mutations in several cancer genes were subsequently identified in these regions: ATM and BIRC3 in 11q or *TP53* in 17p. Sanger sequencing as well as fluorescence *in situ* hybridisation or genomic arrays have further identified a wide spectrum of genomic modifications emphasising the marked genetic heterogeneity of CLL.

Considerable progress has been made in the field of CLL genetics over last 3 years, with the publication of multiple studies using next generation sequencing (NGS) culminating with two recent reports in *Nature*, which describe the whole exome/genome sequencing of 990 CLL patients.^1, 2^ Both studies confirmed the considerable genetic heterogeneity of CLL as well as its evolution during disease progression.

Tumour genome sequencing shatters genetic information into small pieces, which must be then reconstructed like a giant jigsaw puzzle without a picture on the box. Assembling genetic alterations, based on previous knowledge about biological processes, allows investigation of entire sets of mutations in a tumour and determination of the targeted pathways. The fundamental goal is to decrease the data set concerning countless altered genes and proteins to a smaller and more meaningful set of altered pathways. This strategy will generate testable hypotheses, identify tumour subtypes with clinically distinct outcomes, characterise the cancer-specific and cross-cancer pathways and help to identify drug targets. Furthermore, analysis of large cohorts of patients has revealed that the driver mutations targeting multiple components of a single pathway are mutually exclusive on tumour samples. Identification of this type of exclusive pattern is important for the understanding of cancer progression, leading to a stimulating feedback between the patient and the lab, as this analysis may lead to the proposal of genes for targeted therapy.

The *TP53* network is a paradigm for this type of analysis, as it is impaired in most human cancers.^3^ The backbone of this pathway is the *TP53* autoregulatory feedback loop and its negative regulator MDM2 (Figure 1). Depending on the type of stress, multiple upstream signals can disrupt this regulation leading to *TP53* activation and initiation of a complex transcriptional program, which is essential to maintain cellular homoeostasis.

Inactivation of several members of this network in CLL has already been clearly established with an apparent focus on the DNA-damage pathway with ATM and POT1 mutations (Figure 1). Although the mutual exclusivity of ATM and *TP53* alterations has already been reported, the observation that, in the series of 58 POT1 mutations reported by Puente *et al.* and Landau *et al.*, only one patient also harboured a *TP53* mutation is a strong argument to include POT1 alterations in the *TP53* network targeted in CLL. POT1 is an essential component of shelterin, a protein complex that shapes and safeguards human telomeres and activates the *TP53* pathway via ATR kinase, inducing telomere shortening or uncapping, and therefore, preventing chromosomal instability. Whether or not these tumours exhibit a particular genetic instability is currently unknown. The link between POT1 and *TP53* is reinforced by the recent finding of POT1 germline mutations in three *TP53*-negative Li–Fraumeni-like families with cardiac angiosarcoma, a very rare malignant tumour.^4^ A few ATR mutations have also been described in CLL, but it is unclear whether they are mutually exclusive to POT1 (and ATM) mutations.

RPS15 mutations shed light on another aspect of the *TP53* pathway (Figure 1). Accurate ribosome biogenesis is carefully controlled to prevent quantitative and qualitative protein translation.^5^ The MDM2 protein is critical for this nucleolar response via binding of 5S RNP, which contains 5SRNA, RPL11 and RPL5 in response to impaired ribosomal biogenesis.^6^ More recently, other proteins associated with the small subunit of the ribosome (RPS15 or RPS30) have been shown to bind and inactivate MDM2, leading to a strong *TP53* response and cell death.^7^ It has been hypothesised that RPS15 (like several other ribosomal proteins) could act as a ‘detector' of impaired ribosomal biogenesis, explaining why RPS15 mutations can contribute to tumourigenicity. Although only a small number of patients harbour RPS15 mutations, these mutations tend to be exclusive of *TP53* alterations and are associated with shorter progression-free survival (PFS).

In a seminal paper, Rossi *et al.*^8^ demonstrated BIRC3 inactivation in a subset of patients who were refractory to fludarabine therapy. This observation was confirmed by the work of Landau *et al.* and Puente *et al.*, who both showed that BIRC3 mutations are associated with shorter survival, a feature shared by patients with ATM or *TP53* alterations. A remarkable feature of BIRC3 mutations is their occurrence in tumours not presenting any *TP53* mutations, suggesting that they are associated with a common pathway. BIRC3, also known as cIAP2 (cellular inhibitor of apoptosis proteins), is a regulator of canonical NF-kB signalling downstream from the TNF-R1 receptor, and also functions as a negative regulator of the non-canonical NF-kB pathway via RING finger domain-dependent ubiquitination of NIK. In a cellular model, downregulation of BIRC3/cIAP2 led to TP53 degradation via NF-KB-dependent phosphorylation and activation of mdm2.^9^ On the other hand, most BIRC3 mutations are localised in the carboxy terminus, resulting in proteins that are deficient for their ubiquitination activity, suggesting a possible gain of function.

Despite the multiple links between the *TP53* and NF-kB pathways, the mutually exclusive nature of BIRC3 and *TP53* mutations cannot be easily explained, but should be explored in more detail to gain insight into the mechanisms, leading to resistance to therapy.

MicroRNAs are an important component of the BCR (B-cell receptor) signalling pathway. The signature profile of microRNAs can distinguish normal B cells from malignant CLL. Several microRNAs regulated by TP53, such as miR-15a, miR-161 localised on chromosome 13 or mi-R34A/b localised on chromosome 11, are frequently deregulated in CLL. Whether or not, these defects impair the TP53 pathway currently remains unknown, but should be investigated to gain more insight into the role of microRNA defects.

Finally, a few words on *TP53* mutations. Although seemingly infrequent in the early phase of the disease, *TP53* mutations are often found in patients with relapse/refractory disease and poor survival.^10^ Nonetheless, deep NGS has demonstrated the presence of very small subclones in upto 9% of cases at diagnosis, and retrospectively in early samples of patients who exhibited larger clones at later stages of progression.^11^ It is noteworthy that approximately one half of these patients did not harbour a 17p deletion, and therefore, retained the wild-type *TP53* allele. It is currently unknown whether *TP53* mutations observed in patients with and without 17p deletion have different biochemical properties. The importance of TP53 during disease progression is also emphasised by the identification of multiple subclones harbouring different *TP53* mutations in leukaemic cells. The presence of *TP53* mutation has been linked to chemorefractoriness, and expansion of *TP53*-mutated subclones has been clearly demonstrated in patients relapsing after initial chemotherapy.

Defining the origin of these clones, how they evolve and perhaps compete during tumour progression, are among the several questions that NGS will be able to resolve in the near future.

The presence of *TP53* mutations represent an adverse prognostic parameter at all time points of CLL history: at diagnosis, at the time of initial therapy and at relapse. New agents such as BCR inhibitors have provided very promising results, and have been approved by both FDA and EMA for use as first-line therapy in the presence of *TP53* alteration. It is therefore highly recommended to test patients for the presence of 17p deletion and *TP53* mutation before treatment decisions, even beyond the context of clinical trials.

Clearly, several upstream pathways converging to *TP53* are impaired in CLL, as well as alteration of the *TP53* gene itself. Whether or not this can be considered as a single clinical entity, has to be resolved yet. The observation that ATM, RPS15, *TP53* or BIRC3 alterations are associated with shorter overall survival and PFS is strong argument in favour of this hypothesis, which is reinforced by the observation that *TP53* mutations are strongly exclusive with BIRC3, ATM and RPS15 mutations in the series of 990 cases of CLL published by Landau *et al.* and Puente *et al.* Although the exclusive nature with ATM mutations is not always observed, the discovery that CLL, and more particularly cases of CLL in progression or relapse, display high levels of subclones that could harbour different genotypes could explain this apparent discrepancy. Analysis of clonal evolution as well as single-cell sequencing will be able to refine our knowledge on the genetics of CLL.

## Figures and Tables

**Figure 1:**
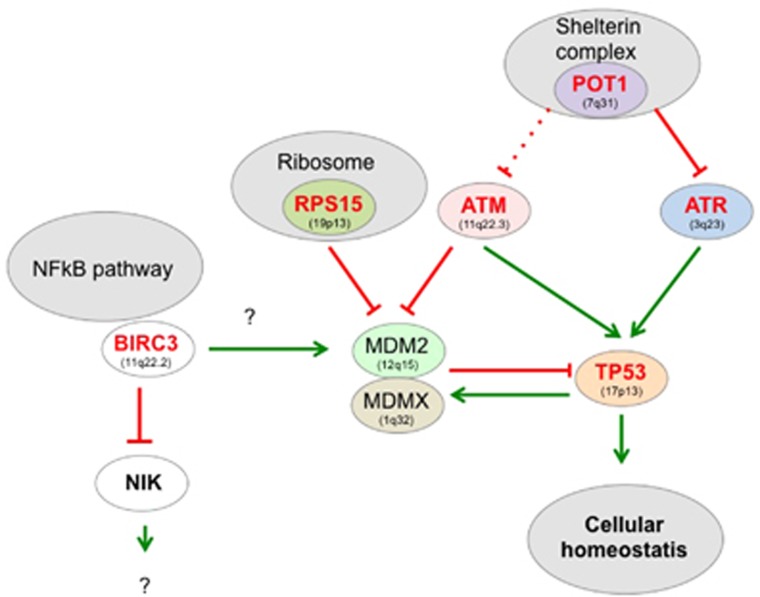
The *TP53* pathway in CLL. The level of TP53 protein is downregulated via binding of proteins, such as, MDM2 that promote TP53 degradation via the ubiquitin/proteasome pathway. As MDM2 is upregulated by TP53, this leads to a regulation loop, which maintains a very low level of TP53 protein in normal cells. This function also requires MDMX, which binds both MDM2 and *TP53*. Disruption of this equilibrium leading to TP53 accumulation is the main outcome of the upstream pathways to promote a *TP53* biological response. In CLL, two pathways associated with DNA damage (double-strand breaks or telomere uncapping) are clearly impaired. Genes depicted in red are impaired in CLL. Whether or not trisomy 12 can alter the *TP53* pathway by increasing mdm2 activity is controversial. The links between BIRC3 and the *TP53* pathway remain elusive
